# Effect of different thermal stimuli on improving microcirculation in the contralateral foot

**DOI:** 10.1186/s12938-021-00849-9

**Published:** 2021-02-02

**Authors:** Weiyan Ren, Liqiang Xu, Xuan Zheng, Fang Pu, Deyu Li, Yubo Fan

**Affiliations:** 1 Key Laboratory of Rehabilitation Technical Aids of Ministry of Civil Affair, School of Biological Science and Medical Engineering, Beihang University, Beijing, People’s Republic of China; 2 State Key Laboratory of Virtual Reality Technology and Systems, Beihang University, Beijing, People’s Republic of China; 3 Beijing Key Laboratory of Rehabilitation Technical Aids for Old-Age Disability, Key Laboratory of Human Motion Analysis and Rehabilitation Technology of the Ministry of Civil Affairs, National Research Center for Rehabilitation Technical Aids, Beijing, People’s Republic of China; 4 School of Biological Science and Medical Engineering, Beihang University, No.37 Xueyuan Road, Haidian District, Beijing, 100191 People’s Republic of China

**Keywords:** Microcirculation disorders, Contralateral thermal effect, Warm bath, Infrared radiation, Plantar foot

## Abstract

**Background:**

The lower extremities of the body often suffer from impaired microcirculation, particularly in the elderly or people with underlying conditions such as diabetes. Especially for people suffering from peripheral vascular diseases, skin lesions or wearing an external fixator in one side of limbs, direct contact treatments are not suitable for them to improve microcirculation. Heating the contralateral limb has been reported to improve blood flow in the impaired limb. However, its effect on plantar microvascular responses has not been previously investigated. Thus, the aim of this study was to explore how heating by warm bath and infrared radiation affects the circulations in the contralateral foot. Twelve healthy adults participated in this study and were randomly assigned to either placing the left foot in a warm bath or exposing it to infrared radiation for 10 min intervention every other day. The skin temperature (Temp) and skin blood flow (SBF) in the second metatarsal head of the contralateral foot were measured before and after the intervention.

**Results:**

The results showed that both Temp (Bath: from 29.05 ± 3.56 °C to 31.03 ± 4.14 °C; Infrared: from 29.98 ± 3.86 °C to 31.07 ± 3.92 °C) and SBF (Bath: from 62.26 ± 48.12 PU to 97.76 ± 63.90 PU; Infrared: from 63.37 ± 39.88 PU to 85.27 ± 47.62 PU) in the contralateral foot were significantly increased after heating in both tests (*p* < 0.05). However, the contralateral SBF increased for 5 min after heating in warm bath test, but only for 1 min in infrared radiation test.

**Conclusions:**

The results of this study show that both heating methods are the effective at increasing contralateral Temp and SBF, but the warm bath has a stronger residual thermal effect.

## Background

Insufficient blood supply often occurs in the lower extremities in people with microcirculation disorder, especially in people with diabetes [1, 2]. Poor circulation in the foot can cause ischemia and hypoxia of the foot tissue, and also affect the neural nutritional supply. The reduced sensory perception to external stimuli and impaired nerve regulation of microvasculature will increase the risk of developing foot lesions and ulcers, which directly affects the life quality and health of people [3–5].

Clinical methods to treat dysfunction microcirculation include drugs [6] and physical agents (e.g., heating [7–9], mechanical stimulations [10, 11], extracorporeal shock wave [12], electromagnetic field stimulation [13], transcutaneous electrical nerve stimulation [14], etc.). As a convenient, safe and effective method, heating is often used in clinical treatment and daily life to promote blood circulation, nutritional supply, wound healing and tissue repair [15, 16]. However, for people suffering from peripheral vascular diseases, skin lesions or wearing an external fixator, it is often unsuitable to apply a thermal stimulus directly to the affected area. In such instances, an effective method to improve blood supply insufficiency in the affected limbs is necessary for people with microcirculation disorder.

The study of Kubo et al. pointed out that applying heating treatments or acupuncture to the unaffected tendon of contralateral limb can increase the blood volume in the injured tendon in a plaster cast [17]. Astrup et al. found that contralateral heating can increase the subcutaneous blood flow of the unheated forearm and affect metabolism of the unheated hand [18]. And Gorodkin et al. reported that the vasodilator responses to heating stimulus were similar in the affected and unaffected areas of calf in people with complex regional pain syndrome [19]. These previous studies have demonstrated that applying heating to the contralateral side of the limb can improve the blood circulation in the unheated side of the limb. However, the distribution of the microvascular network in the foot and its regulation mechanism are different from other parts of the body [20, 21]. The influence of heating intervention on the foot tissue still needs to be further explored.

Warm bath is a common and safe method for promoting blood circulation in the routine clinics and home therapy [22], which can transfer heat directly to the skin tissue via water medium, and induce cutaneous vasodilation by stimulating peripheral and core thermoreceptors [23]. Moreover, infrared radiation is often used as a clinical treatment method to accelerate peripheral circulation because of its convenient and effective features [8, 24], which can induce heat-related and non-heat-related biological effects and elicit vasodilatation by transferring energy into heat and causing molecular resonance [25, 26]. Kim and Marshall et al. reported that, a warm bath is an effective method to evoke reflexive microvascular vasodilation in the contralateral hand and mid-forearm of healthy people [23, 27]. The experimental results of the study of Rutkowski et al. showed that infrared radiation could increase the skin temperature in the contralateral dorsal side of hand by heating the other hand in people with rheumatoid arthritis [28]. However, previous studies tended to focus on the thermal effects in the upper limbs, but for human foot that often suffers from microcirculation disorders, few studies have reported the heating effects on it [20]. Thus, exploring the effects of warm bath and infrared radiation on improving microcirculation in contralateral foot may be helpful in developing protection methods for people with foot microcirculation disorders.

This study used either a water bath or infrared radiation to heat one foot of each subject and recorded the cutaneous temperature and blood flow responses in the contralateral foot. The aim was to compare and understand the differences in contralateral thermal effects of the two heating methods. We hypothesize that compared to infrared radiation, warm bath may induce a greater contralateral microvascular response for its prolonged effects on the regulation of peripheral and core thermoreceptors.

## Results

The results of normality analysis shows that, the Temp and SBF data in two tests did not satisfy the normal distribution, thus a Wilcoxon matched-pair signed-rank test was used to test the difference for Temp and SBF response between pre- and post-intervention.

**Fig. 1:**
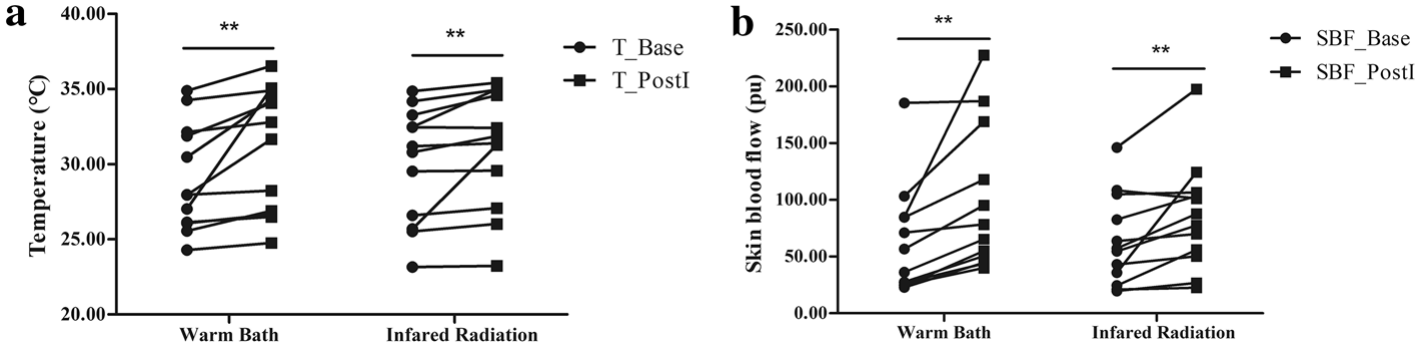
The plantar temperature (**a**) and skin blood flow (**b**) before and immediately after heating. T_Base and SBF_Base indicate the mean skin temperature and skin blood flow in the baseline stage, respectively. T_ PostI and SBF_PostI indicate the mean skin temperature and skin blood flow in the Recovery stage during the first 10 s after heating. **Indicates a significant difference between the Baseline stage and Recovery stage, *p* < 0.01

The Temp and SBF of M2 in the subjects’ right feet before and immediately after thermal stimuli are shown in Fig. 1. In the warm bath test, there was a significant increase in mean instant Temp from the Baseline (29.05 ± 3.56 °C) to post-intervention (31.03 ± 4.14 °C), and the mean instant SBF increased significantly from 62.26 ± 48.12 PU to 97.76 ± 63.90 PU. Similarly in the infrared radiation test, the mean instant Temp increased significantly from a Baseline temperature of 29.98 ± 3.86 °C to a post-intervention temperature of 31.07 ± 3.92 °C, and the mean instant SBF increased significantly from 63.37 ± 39.88 to 85.27 ± 47.62 PU. Moreover, the results of power analyses of the instant skin temperature and skin blood flow responses showed that, 100% power was achieved based on the selected sample size to detect the contralateral thermal effects induced by the two heating methods with a significance level of 0.05. These results indicate that both a warm bath and infrared radiation can increase the cutaneous temperature and blood flow in the contralateral foot.

**Fig. 2:**
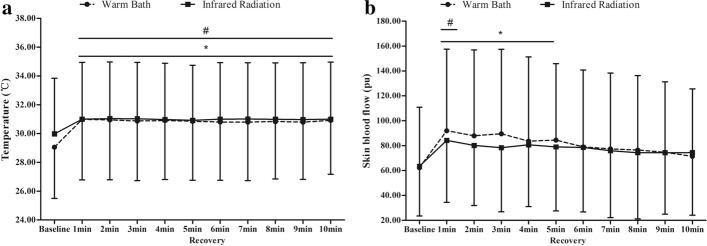
The plantar temperature (**a**) and skin blood flow (**b**) during the Baseline and Recovery stage. *Indicates a significant difference between the Baseline and Recovery stages of the warm bath test; *p < 0.05. ^#^Indicates a significant difference between the Baseline and Recovery stages of the infrared radiation test; ^#^*p* < 0.05

Figure 2 shows the mean Temp and SBF of M2 in the subjects’ right feet during the Baseline stage and for the duration of the Recovery stage. It can be seen that for both tests the Temp was significantly higher during the Recovery stage than in the Baseline stage. However, while the SBF increased significantly for the initial 5 min of the Recovery stage in the warm bath test, there was only a significant increase for the first 1 min of the infrared radiation test. These results indicate that both two heating methods have a thermal effect on the contralateral foot, but the warm bath has a stronger residual effect. Comparison results for the cutaneous temperature and blood flow of Recovery stage to Baseline stage in two tests are shown in Table 1.

**Table 1:** Comparison results for cutaneous temperature and blood flow of Recovery stage to Baseline stage in two tests

	Warm bath			Infrared radiation		
	*p* value	95% confidence interval of the difference	Effect size	*p* value	95% confidence interval of the difference	Effect size
Recovery stage						
Temperature						
Instant(0–10 s)	0.002	− 3.27 ~ − 0.69	0.87	0.004	− 1.99 ~ − 0.19	0.69
1 min	0.002	− 3.29 ~ − 0.58	0.81	0.010	− 1.9 ~ − 0.14	0.66
2 min	0.002	− 3.26 ~ − 0.53	0.78	0.004	− 1.92 ~ − 0.19	0.69
3 min	0.002	− 3.2 ~ − 0.45	0.75	0.004	− 1.98 ~ − 0.13	0.65
4 min	0.002	− 3.2 ~ − 0.5	0.78	0.005	− 1.9 ~ − 0.09	0.62
5 min	0.002	− 3.13 ~ − 0.46	0.76	0.004	− 1.86 ~ − 0.03	0.58
6 min	0.002	− 3.07 ~ − 0.42	0.74	0.004	− 1.93 ~ − 0.11	0.64
7 min	0.002	− 3.08 ~ − 0.39	0.73	0.005	− 1.99 ~ − 0.07	0.61
8 min	0.003	− 3.11 ~ − 0.45	0.76	0.008	− 2 ~ − 0.03	0.58
9 min	0.003	− 3.09 ~ − 0.39	0.73	0.013	− 1.95 ~ − 0.03	0.58
10 min	0.003	− 3.19 ~ − 0.51	0.78	0.015	− 2.06 ~ 0.02	0.55
Skin blood flow						
Instant(0–10 s)	0.003	− 56.75 ~ − 13.93	0.93	0.010	− 37.03 ~ − 6.77	0.82
1 min	0.008	− 52.21 ~ − 6.88	0.74	0.023	− 36.46 ~ − 5.22	0.75
2 min	0.008	− 47.66 ~ − 3.25	0.65	0.050	− 31.22 ~ − 2.41	0.66
3 min	0.002	− 49.12 ~ − 5.04	0.70	0.060	− 31.56 ~ 1.7	0.51
4 min	0.028	− 42.62 ~ − 0.12	0.57	0.006	− 38.8 ~ − 5.38	0.82
5 min	0.005	− 39.27 ~ − 4.68	0.72	0.060	− 31.9 ~ 0.68	0.54
6 min	0.099	− 36.06 ~ 2.98	0.48	0.084	− 30.79 ~ 0.35	0.55
7 min	0.182	− 31.92 ~ 2.17	0.49	0.117	− 29.43 ~ 4.26	0.42
8 min	0.272	− 30.75 ~ 2.68	0.48	0.433	− 28.76 ~ 6.84	0.35
9 min	0.433	− 28.8 ~ 4.48	0.41	0.754	− 29.85 ~ 8.11	0.32
10 min	0.480	− 24.73 ~ 6.91	0.32	0.937	− 31.04 ~ 9.03	0.31

## Discussion

This study heated the left feet of subjects using two different thermal stimuli in order to investigate their effects on cutaneous temperature and blood flow responses in the contralateral plantar foot. The results showed that both the warm bath and infrared radiation increase the cutaneous temperature and blood flow in the contralateral foot, and the warm bath has a stronger residual thermal effect.

Studies have shown that physiological thermoregulation is mainly controlled by the preoptic/anterior hypothalamus (POAH), which regulates vasodilation and vasoconstriction of blood vessels by the internal and/or skin temperature [29]. When applying a thermal stimulus to one side of the foot, the skin thermoreceptors in the heated area will signal the POAH and activate temperature-sensitive neurons. In the efferent pathway, the projections from the rostral ventrolateral medulla and hypothalamus to the intermediolateral column of the spinal cord are predominantly ipsilateral, but also produce contralateral signals. Thus, the contralateral thermoregulatory effectors will also receive signals to induce skin vasodilation [30]. Cranson et al. reported that the temperature of arterial blood flow increased by 0.5 °C within 4 min when the forearm was placed in water at 40 °C [31]. The increased temperature of arterial blood flow would activate the central thermal receptors, consequently inducing vasodilation of bilateral skin vessels and increasing peripheral blood flow and cardiac output [32, 33]. Thus, a thermal stimulus applied to one side of the foot can induce contralateral responses in skin temperature and blood flow by the innervation of peripheral and central thermal receptors.

The plot in Fig. 2 shows that the residual effects of the two thermal stimuli on contralateral skin blood flow are different. The skin blood flow of M2 in the contralateral foot was significantly higher than the basal blood flow for 5 min after removal from the warm bath, while the skin temperature was only significantly higher for the first 1 min after removing the infrared radiation heat. This indicates that heating the foot with a warm bath leaves a stronger residual contralateral effect than infrared radiation, which may be due to the different contralateral regulatory mechanisms between the two thermal stimuli.

Marshall et al. reported that if one hand was heated by a warm bath for 2 min, blood flow in the contralateral hand would increase, and there would be a further increase in blood flow and cardiac output after 5 min of heating [23]. The elevated cardiac output during thermal stimulation is primarily caused by the increase in internal temperature [34]. This suggests that the reflexive vasodilation of skin vessels in the contralateral limb during heating is not only mediated by skin thermoreceptors in the heated limb, but also by the central thermoreceptor activated by the increased temperature of arterial blood flow. When the source of heat is removed, although the stimulus to the skin thermoreceptors ceases and afferent signals to the POAH are greatly reduced, the internal temperature of arterial blood would not drop immediately, which would take longer for the blood perfusion and blood temperature to recover to baseline values because the heat needs to be dissipated though vessel vasodilation. Thus, in this study, heating in a warm bath for 10 min may stimulate both the peripheral and central thermoreceptors by increasing the temperature of skin and arterial blood flow, resulting in a longer recovery time and a stronger residual effect on subjects’ contralateral feet.

When soft tissue is heated by infrared radiation, heat will be generated by resonance and friction between molecules because the vibration frequency of partial far infrared rays is close to that of intracellular molecules in soft tissue, which causes an increase in skin temperature and accelerates blood circulation [8, 35]. The thermal effect in the heated side of limbs will be regulated by the thermoregulation of central nervous systems, making the temperatures in the contralateral side and the irradiated side close [36]. Another theory is that, the infrared radiation can induce the release of cytokines and growth factors in the circulation, contributing to the vasodilation of ipsilateral and contralateral vessels [28, 37]. However, the far infrared rays can only be transmitted into subcutaneous tissue in a depth of 2–3 mm [38], and may have a relatively slight effect than warm bath, which can induce vessels vasodilation regulated by both peripheral and central thermoreceptors [23, 34].

Another reason for the different residual effects may be the conducting medium. Water is a good conductor and can transfer heat to the whole foot. When heating with infrared radiation, the stagnant air may attenuate heat from radiator to plantar foot [39]. To mitigate the difference in the heat transferred to the foot, the temperature of the measurement areas under infrared light was ensured to be 40 °C by controlling the device.

The second metatarsal head (M2) typically experiences high plantar pressure when walking and, as such, is a common site for foot ulcers in diabetics with impaired microcirculation [40]. Thus, this study chose M2 to investigate the thermal effects of two heating methods. Moreover, since 10 min of thermal stimulation can transmit heat stress into skin tissue to a depth of 1 cm [15, 41], and a thermal stimulus with a temperature below 40 °C is generally safe [38], this study used these parameters to achieve a thermal effect and avoid skin burns.

In conclusion, this study found that both the warm bath and infrared radiation are effective at increasing the temperature and skin blood flow in the contralateral foot, but there are differences in the residual effects. Heating with infrared radiation has been used to treat microvascular dysfunction, sports injuries, etc. [25, 42], which is generally more convenient to use, and is more suitable for detecting nerve and vascular function because of the instantaneous change in temperature and blood flow in the contralateral limb. The warm bath is widely used in the clinical and home therapy [22], and has a longer lasting thermal effect, and may be more suitable for use by people who suffer from skin lesions or wear an external fixator, and cannot accept the heat source being focused on one side of the limb.

This study has some limitations that should be noted. The temperature of both warm bath and infrared radiation was set at 40 °C, but the power produced by the two sources has not been controlled, which needs to be further verified in future research. Moreover, this study only considered heating the feet of healthy subjects. Future studies may consider other limbs like dominant side or enrolling subjects with impaired microcirculation. In addition, although the fluctuation range of SBF data is consistent with the results of other studies [43, 44], the standard deviation of SBF results is slightly large due to the individual differences. In further studies, the characteristics of basal SBF should be considered when screening subjects.

## Conclusions

This study explored how heating the left foot with a warm bath and infrared radiation affected the skin temperature and blood flow in the contralateral foot. The results showed that both heating methods can effectively increase the cutaneous temperature and blood flow in the contralateral foot, but the warm bath produced a longer duration of cutaneous vasodilation.

## Methods

### Study design

This study is a repeated measures observation study designed to analyze the effects of water bath and infrared radiation interventions on cutaneous temperature and blood flow responses in the contralateral foot.

### Participants

Seven males and five females (23.50 ± 0.52 years, 20.70 ± 2.11 kg/m^2^) were recruited in this study. The inclusion criteria were: (1) aged from 20 to 25 years old with the body mass index from 18.5 to 23.9 kg/m^2^; (2) had no symptoms such as redness, callus, inflammation, or wounds on the skin of the feet or legs, and (3) had no diseases such as hypertension, peripheral neuropathy, vascular disease, heart disease, systematic inflammation, malignant tumor, etc. According to the estimation of sample size based on the microcirculation results of contralateral unheated limb in previous study [28], the sample size in this study is adequate for statistical analysis. This study was conducted in accordance with clinical protocols approved by the institutional review board of Affiliated Hospital of National Research Center for Rehabilitation Technical Aids and in accordance with the Declaration of Helsinki. All subjects gave informed written consent prior to participation.

### Procedures

Before the test, all subjects were asked to maintain a sitting position with legs straight and bare feet for 30 min in a room at a temperature of 25 ± 2 °C, in order to acclimate to the experimental environment and keep a stable state [11]. Considering the second metatarsal head (M2) is the area with high-risk of foot tissue ischemia [40], it was selected as the region of interest for measuring microcirculation parameters. To set a baseline, the skin temperature (Temp) and skin blood flow (SBF) of M2 in each subject right foot were measured for 5 min (Baseline stage). The left foot was then heated for 10 min using either a warm bath or infrared radiation randomly (Intervention stage) [15, 41]. After heating, the Temp and SBF of M2 in the right foot were measured again for 10 min (Recovery stage). Temp was measured using a thermal infrared imager (TiS45, Fluke, United States), and SBF was measured by a laser speckle contrast imaging flowmeter (PeriCam PSI System, Perimed, Sweden). All subjects were arranged to accept warm bath and infrared radiation interventions, and the heating method (bath or infrared) was randomly applied to each subject at the same time in 2 days.

An adjustable foot tub (DZ 8861, Dongzhi, China) was used to provide warm bath for the foot. The water temperature of the warm bath was controlled at 40 ± 1 °C, and the water level reached the medial malleolus of the foot [31]. Two adjustable infrared lamps (E27 100W, Philips, Poland) were used to provide infrared radiation for the plantar foot and dorsum foot, respectively. The radiator was placed 20 cm away from subjects’ feet, and the temperature emanating on the skin of feet was also controlled at a safe threshold of 40 ± 1 °C [38], which was achieved by adjusting the button of radiator, and being measured and calibrated by a temperature sensor (TH20R, Miaoxin, China) before tests.

### Data and statistical analysis

The Temp and SBF were recorded during the Baseline stage and averaged to give a mean basal value (T_base, SBF_base). For the Recovery stage, the instantaneous values of Temp and SBF in the first 10 s were averaged to give mean values immediately after heating (T_postI, SBF_postI). For the remaining 10 min of the Recovery stage, the mean values of Temp and SBF were calculated for every minute (T_post_1-10 min_, SBF_post_1-10 min_).

A Shapiro–Wilk test was used to test the normality of the variables. If the variables were normally distributed, a paired t-test was used to test the difference in Temp and SBF responses between pre- and post-intervention. If the variables were not normally distributed, a Wilcoxon matched-pair signed-rank test was used. A statistical significance level of 0.05 was used. All statistical analyses were performed in SPSS (Version 22.0, IBM, Armonk, NY, USA).

## Data Availability

All data generated or analyzed during this study are included in this published article.

## Abbreviations

Temp: Temperature
SBF: Skin blood flow
M2: The second metatarsal head
POAH: Preoptic/anterior hypothalamus
T_base: The mean value of temperature during the Baseline stage
SBF_base: The mean value of skin blood flow during the Baseline stage
T_postI: The mean values of instantaneous temperature in the first 10 s of the Recovery stage
SBF_postI: The mean values of instantaneous skin blood flow in the first 10 s of the Recovery stage
T_ post1-10min: The mean values of temperature in each minute during the 10 min of the Recovery stage
SBF_post1-10 min: The mean values of skin blood flow in each minute during the 10 min of the Recovery stage
